# Early Detection of Acute Myocarditis in the Pediatric Population Using Clinically Accessible Data

**DOI:** 10.1111/ped.70492

**Published:** 2026-07-27

**Authors:** Takahiro Motonaga, Seigo Okada, Yuji Ohnishi, Takashi Furuta, Shunji Hasegawa

**Affiliations:** ^1^ Department of Pediatrics Yamaguchi University Graduate School of Medicine Yamaguchi Japan

**Keywords:** acute myocarditis, early diagnosis, gallop rhythm, pediatric population, serum lactate dehydrogenase

## Abstract

**Background:**

Early diagnosis of acute myocarditis (AMC) is crucial due to its rapid progression and high mortality rate. Initial symptoms such as fever, abdominal pain, and vomiting are nonspecific and pose challenges for clinicians in distinguishing AMC from acute gastroenteritis (AGE).

**Methods:**

We retrospectively reviewed the clinical characteristics of seven patients with AMC who were hospitalized at our institution between April 2011 and March 2022. We compared these findings with those of 19 patients with AGE.

**Results:**

The common initial symptoms of patients with AMC included fever, vomiting, abdominal pain, appetite loss, nausea, and fatigue, which did not differ significantly from those in patients with AGE. However, the percentage of diarrhea was significantly lower, whereas the detection rates of tachypnea, tachycardia, gallop rhythms, or cardiomegaly on radiography were higher in the patients with AMC than in those with AGE, respectively. Additionally, patients with AMC exhibited significantly higher serum lactate dehydrogenase (LDH) levels than those with AGE (*p* = 0.006). LDH demonstrated 71% sensitivity and 100% specificity (area under the curve, 0.857) for predicting AMC when a cutoff value of 459 U/L was used.

**Conclusions:**

Preliminary findings suggest that LDH may serve as a trigger for suspicion of AMC in the pediatric population. Furthermore, combining LDH results with physical examination and chest radiographic findings could be useful for early diagnosis.

## 1 Introduction

Acute myocarditis (AMC) is characterized by sudden myocardial inflammation and rapid disease progression [1]. The clinical manifestations of AMC vary from subclinical to fulminant, with a high mortality rate [2, 3, 4]. AMC is considered a medical emergency requiring immediate and intensive medical intervention. In particular, for fulminant myocarditis (FMC), early diagnosis and the introduction of mechanical circulatory support, such as extracorporeal membrane oxygenation, are important because of the high mortality rate [4]. Mechanical circulatory support may be a successful strategy for supporting patients until cardiac dysfunction resolves or as a bridge to transplantation, as 76% of high‐risk patients are successfully bridged to transplantation or recovery [5].

Viral infections and/or post‐viral immune‐mediated responses are among the most common etiologies of AMC during childhood [2]. In a previous surveillance study, pathogens were identified in 37 (21.9%) of 169 children, with coxsackie viruses accounting for 60% [4]. Coxsackie virus infections are a common cause of gastroenteritis in children [6]. It is challenging for clinicians to distinguish between gastroenteritis and AMC in the early stages because AMC often presents with nonspecific symptoms, such as fever (47.9%), nausea or vomiting (30.2%), cough (16.6%), abdominal pain (15.4%), and diarrhea (7.7%) [4]. Several reports and reviews have described cases in which children with AMC were initially misdiagnosed as having acute gastroenteritis (AGE), leading to delayed diagnosis [7, 8]. Therefore, AGE is considered an important differential diagnosis. Pathological confirmation of myocardial inflammation is required for a definitive diagnosis of AMC [9]. Cardiac magnetic resonance imaging is currently the most useful imaging tool for AMC diagnosis [9]. However, in clinical settings, myocardial biopsy or cardiac magnetic resonance imaging is often difficult to perform during the acute phase because of the unstable condition of pediatric patients. Accordingly, a combination of additional clinical assessments—including physical examination of the chest, electrocardiography (ECG) [9, 10], ultrasonography [9], and blood tests for brain natriuretic peptide (BNP), creatine kinase (CK), and troponin T (TnT) [9]—is generally used to establish a diagnosis of myocarditis. However, none of these has been established as a predictive tool for AMC. Most patients with AMC present to general pediatric clinics or emergency departments (ED) for their initial consultation. A previous report from an Italian group highlighted the utility of ECG as a simple bedside tool to support early suspicion of AMC in the ED and to guide appropriate diagnostic testing [10].

Although ECG and the aforementioned cardiac biomarkers are considered useful in diagnosing or detecting AMC at an early stage, they are not always performed in clinical settings where children are often unable to clearly describe their symptoms, and attending physicians may not be cardiology specialists. Based on this background, we aimed to develop an effective predictive tool for AMC using data from routine medical practice.

## 2 Materials and Methods

### 2.1 Study Design and Subjects

We performed a retrospective database review and subsequent outcome analysis. We enrolled children who presented with nonspecific symptoms, such as fever, vomiting, and abdominal pain and were ultimately diagnosed with either AMC or AGE. We enrolled seven consecutive patients with AMC, including four patients with FMC, who were admitted to the Yamaguchi University Hospital between April 2011 and March 2022. We consecutively collected data from patients hospitalized in our department with AGE between 2009 and 2016. These included cases of bacterial enteritis, in which the causative pathogen was identified, and cases of viral gastroenteritis, diagnosed based on antigen testing and clinical findings. Patients with missing clinical data were excluded. A cohort of 19 patients with AGE, whose serum had been stored for cytokine measurement during the same period, served as the control group. This group comprised 11 cases of viral gastroenteritis and eight cases of bacterial enteritis. Both bacterial and viral gastroenteritis present with similar gastrointestinal symptoms, such as vomiting, abdominal pain, and diarrhea, making them difficult to distinguish based solely on clinical presentation. Although rare, several cases of myocarditis associated with bacterial enteritis, such as *Campylobacter* spp. and *Salmonella* spp., have also been reported [11, 12]. Accordingly, both viral and bacterial cases were included in the AGE group.

Furthermore, we performed a comparative analysis to distinguish AMC from AGE, both of which share similar initial symptoms and therefore require differentiation. The outcome was defined as the early diagnosis of AMC, and by comparing the clinical presentations with the AGE group, we evaluated whether any parameters could provide new preliminary insights into its early detection. Medical records were retrospectively reviewed to investigate the clinical and laboratory findings of patients at admission. The clinical characteristics included patient age, sex, initial symptoms before admission, physical examination findings at admission, date of first visit, diagnosis, treatment, and prognosis. Laboratory findings included blood examination, ECG, chest and/or abdominal radiography, and echocardiography at admission. The contents of blood examination were as follows: white blood cells (WBC), C‐reactive protein (CRP), aspartate aminotransferase (AST), alanine aminotransferase (ALT), lactate dehydrogenase (LDH), CK, CK‐myocardial band, BNP, TnT, potassium, lactate (Lac), total bilirubin (T‐Bil), prothrombin time‐international normalized ratio (PT‐INR), activated partial thromboplastin time (APTT), fibrinogen (Fib), and D‐dimer. For ECG, radiography, and echocardiography, we examined the presence of ST segment changes and AV block, cardiothoracic ratio > 55%, left ventricular end‐diastolic diameter, left ventricular ejection fraction, presence of pericardial effusion, and atrioventricular regurgitation.

This study was approved by the Institutional Review Board of the Yamaguchi University Hospital. Written informed consent was obtained from the parents of all patients enrolled in this study.

### 2.2 Statistical Analysis

The Wilcoxon rank sum test was used to compare the levels of blood test parameters between the AMC and AGE groups. For the analyses of ECG, radiography, and echocardiogram, the *χ* ^2^ test or Fisher's exact test was performed. In multiple testing, *p*‐values were adjusted using the Benjamini–Hochberg false discovery rate (FDR) procedure. We conducted a correlation analysis to evaluate the effect of age on LDH levels. Spearman's rank correlation coefficient was used, and multivariate correlation analysis of the parameters was performed. Receiver operating characteristic (ROC) curve analysis was also conducted to predict AMC. We performed a statistical comparison of the area under the curve (AUC) values using the DeLong test and additionally provided the corresponding confidence intervals. All statistical analyses were performed using JMP Pro ver.18.0 (SAS Institute Inc., Cary, NC) and EZR (Saitama Medical Center, Jichi Medical University, Saitama, Japan), which is a graphical user interface for R (The R Foundation for Statistical Computing, Vienna, Austria). *p*‐values < 0.05 and FDR‐adjusted *p*‐values < 0.05 were considered statistically significant.

## 3 Results

### 3.1 Study Participants

The clinical characteristics of the patients with AMC are summarized in Table 1. There were seven patients with AMC, comprising four females, one infant, one toddler, and five school‐aged children. The initial symptoms before admission included fever in five patients (71%), vomiting in five patients (71%), abdominal pain in four patients (57%), appetite loss in three patients (43%), nausea in three patients (43%), respiratory distress in three patients (43%), cough in three patients (43%), diarrhea in two patients (29%), fatigue in two patients (29%), headache in two patients (29%), nasal mucus in two patients (29%), convulsion in one patient (14%), palpitation in one patient (14%), and chest pain in one patient (14%). Before admission, all patients with AMC were initially diagnosed with AGE (57%) or common cold (43%) at previous clinics. Physical examination findings on admission included tachycardia in six patients (86%), a gallop rhythm on auscultation in five patients (71%), tachypnea in four patients (57%), pale skin in four patients (57%), peripheral coldness in three patients (43%), edema in one patient (14%), and retraction breathing in one patient (14%). The median number of days from symptom onset to the previous clinic visit was 2 days (range: 1–4 days). The median number of days from symptom onset to final diagnosis was 5 days (range: 3–6 days). Of the four patients (57%) treated with percutaneous cardiopulmonary support, all had FMC.

**TABLE 1:** Clinical characteristics of patients with acute myocarditis.

Variables		
Total number		7
Sex (male/female), *n*		3/4
Age, *n* (%)	Infant	1 (14)
	Toddler	1 (14)
	School‐aged children	5 (71)
Initial symptoms before admission, *n* (%)	Fever	5 (71)
	Abdominal pain	4 (57)
	Nausea	3 (43)
	Vomiting	5 (71)
	Diarrhea	2 (29)
	Fatigue	2 (29)
	Appetite loss	3 (43)
	Convulsion	1 (14)
	Palpitation	1 (14)
	Chest pain	1 (14)
	Respiratory distress	3 (43)
	Headache	2 (29)
	Nasal mucus	2 (29)
	Cough	3 (43)
Initial diagnosis at previous clinics, *n* (%)	Acute gastroenteritis	4 (57)
	Common cold	3 (43)
Physical examination findings at admission, *n* (%)	Tachypnea	4 (57)
	Retraction breathing	1 (14)
	Edema	1 (14)
	Peripheral coldness	3 (43)
	Pale skin	4 (57)
	Gallop rhythm	5 (71)
	Tachycardia	6 (86)
Median number of days from onset to previous clinic visit (range)		2 (1–4)
Median number of days from onset to final diagnosis (range)		5 (3–6)
Median number of days from onset to blood sampling (range)		5 (3–6)

The clinical characteristics of patients with AGE are summarized in Table S1. The control group comprised 19 patients with AGE, including 11 patients (six females) with viral gastroenteritis and eight patients (five females) with bacterial enteritis. The causes of viral gastroenteritis were noroviruses in five patients, rotaviruses in five patients, and undefined in one patient. The causes of bacterial enteritis were *Salmonella* spp. in three patients, *Campylobacter* spp. in two patients, and *enteropathogenic Escherichia coli* (*O‐157*) in three patients.

### 3.2 Clinical Presentations in the AMC and AGE Groups

The clinical presentations of the AMC and AGE groups are shown in Table 2. Given the exploratory nature of this study and the limited sample size, we considered the FDR‐adjusted *p*‐values to provide a more appropriate balance between controlling multiplicity and maintaining statistical power. The reason for choosing FDR correction over Bonferroni correction was that, for some analysis parameters, even though clear visual differences were shown in the box plots (Figure S1), the limited sample size could lead to a Type II error. There were no significant differences in sex between the patients with AMC and those with AGE. There was a significant difference in age between patients with AMC and those with viral gastroenteritis (median, 141 months vs. 14 months [*p* = 0.017]) but not between patients with AMC and those with bacterial enteritis (median, 141 months vs. 91.5 months [*p* = 0.452]). As regards initial symptoms before admission, no significant differences were observed in fever, abdominal pain, appetite loss, nausea, or vomiting between the patients with AMC and those with AGE. However, there was a significant difference in diarrhea prevalence (29% for AMC, 100% for viral AGE, and 88% for bacterial AGE). For physical examination findings at admission, peripheral coldness and pale skin were not commonly described in patients with AGE. The percentages of patients with tachypnea, tachycardia, or gallop rhythms were higher in patients with AMC than in those with AGE (57%, 86%, and 71% for AMC, 0%, 27% and 0% for viral AGE, and 25%, 50% and 0% for bacterial AGE). As for the sensitivity and specificity in the diagnosis of AMC, tachypnea, tachycardia, gallop rhythms, or diarrhea were 57% and 89%, 86% and 61%, 71% and 100%, or 28% and 5%, respectively. Regarding prognosis, one patient (14%) died in the patients with AMC. There were significant differences in the intact survival rates between the groups (AMC vs. viral gastroenteritis [*p* = 0.022] and AMC vs. bacterial gastroenteritis [*p* = 0.025]). Blood samples were collected upon admission to our hospital, which corresponded to the day of diagnosis. Regarding the laboratory findings on admission, there were no significant differences in the serum levels of WBC, CRP, ALT, CK, potassium, Lac, T‐Bil, APTT, Fib, and D‐dimer between the patients with AMC and those with AGE. AST and PT‐INR levels in the patients with AMC were significantly higher than those in the patients with bacterial enteritis but not in the patients with viral gastroenteritis. LDH levels in the patients with AMC were significantly higher than those in the patients with AGE (AMC vs. viral: 792 vs. 316 [*p* = 0.037] and AMC vs. bacterial: 792 vs. 235 [*p* = 0.021]). CK‐myocardial band, BNP, and TnT levels were not assessed in most patients with AGE. Only a few patients with AGE underwent both ECG and echocardiography. The percentage of patients with cardiomegaly on plain radiography (cardiothoracic ratio > 55%) was significantly higher in the patients with AMC than in those with AGE (42% for AMC, 0% for viral AGE, and 0% for bacterial AGE). Furthermore, we conducted a subgroup analysis separating fulminant and non‐fulminant cases (Table S2).

**TABLE 2:** Clinical presentations of the participants.

	AMC group (*n* = 7)	AGE group (*n* = 19)		AMC vs. Viral		AMC vs. Bacterial	
		Viral (*n* = 11)	Bacterial (*n* = 8)	*p*	Adjusted *p*	*p*	Adjusted *p*
Female, *n* (%)	4 (57)	6 (55)	5 (63)	1.000	1.000	1.000	1.000
Age, month (IQR)	141 (29–164)	14 (6–22)	92 (58–118)	0.011	0.017	0.452	0.452
Initial symptoms before admission							
Fever, *n* (%)	5 (71)	8 (73)	7 (88)	1.000	1.000	0.569	1.000
Abdominal pain *n* (%)	4 (57)	9 (82)	7 (88)	0.326	0.489	0.282	0.846
Nausea, *n* (%)	3 (43)	N/A	2 (25)			0.608	0.608
Vomiting, *n* (%)	5 (71)	10 (91)	6 (75)	0.528	1.000	1.000	1.000
Diarrhea, *n* (%)	2 (29)	11 (100)	7 (88)	0.003	0.009	0.041	0.062
Fatigue, *n* (%)	2 (29)	N/A	2 (25)			1.000	1.000
Appetite loss, *n* (%)	3 (43)	9 (82)	6 (75)	0.141	0.423	0.619	0.619
Convulsion, *n* (%)	1 (14)	2 (18)	0 (0)	1.000	1.000	0.467	1.000
Palpitation, *n* (%)	1 (14)	N/A	0 (0)			0.467	0.467
Chest pain, *n* (%)	1 (14)	N/A	0 (0)			0.467	0.467
Respiratory distress, *n* (%)	3 (43)	0 (0)	1 (13)	0.043	0.129	0.282	0.421
Headache, *n* (%)	2 (29)	N/A	3 (38)			1.000	1.000
Nasal mucus, *n* (%)	2 (29)	1 (9)	0 (0)	0.528	0.792	0.200	0.600
Cough, *n* (%)	3 (43)	1 (9)	1 (13)	0.245	0.735	0.282	0.423
Physical examination findings at admission							
Tachypnea, *n* (%)	4 (57)	0 (0)	2 (25)	0.011	0.033	0.315	0.315
Retraction breathing, *n* (%)	1 (14)	0 (0)	0 (0)	0.389	0.467	0.467	0.467
Tachycardia, *n* (%)	6 (86)	3 (27)	4 (50)	0.049	0.147	0.282	0.377
Poor skin turgor, *n* (%)	N/A	2 (18)	2 (25)				
Peripheral coldness, *n* (%)	3 (43)	1 (9)^b^	0 (0)^c^				
Pale skin, *n* (%)	4 (57)	1 (9)^c^	1 (13)^c^				
Abdominal distention, *n* (%)	0 (0)	0 (0)	0 (0)				
Abdominal tenderness, *n* (%)	0 (0)	0 (0)	7 (88)			0.001	0.001
Edema, *n* (%)	1 (14)	N/A	N/A				
Gallop rhythms, *n* (%)	5 (71)	0 (0)	0 (0)	0.003	0.006	0.007	0.007
Cardiac murmur, *n* (%)	0 (0)	0 (0)	0 (0)				
Intact survival, *n* (%)	3 (43)	11 (100)	8 (100)	0.011	0.022	0.025	0.025
Death, *n* (%)	1 (14)	0 (0)	0 (0)	0.389	0.467	0.467	0.467
Blood Examination							
WBC, /μL (range)	10,360 (4480–19,860)	10,830 (4910–16,450)	10,785 (5240–12,270)	0.928	0.928	0.772	0.928
CRP, mg/dL (range)	2.87 (0.01–17.00)	0.41 (0.04–12.00)	5.82 (0.02–30.00)	0.103	0.155	0.224	0.224
AST, U/L (range)	101 (15–2495)	50 (29–69)	28.5 (19–44)	0.051	0.051	0.024	0.036
ALT, U/L (range)	426 (11–2227)	32 (11–112)	15 (8–46)	0.160	0.160	0.049	0.074
LDH, U/L (range)	792 (236–3234)	316 (253–374)	235 (197–297)	0.037	0.037	0.007	0.021
CK, U/L (range)	520 (25–1879)	74 (52–275)	97 (29–606)	0.205	0.615	0.856	0.856
CK‐MB, U/L (range)	32 (7–127)	30 (19–33)^d^	N/A	0.649	0.649		
BNP, pg/mL (range)	914 (550–6485)	N/A	N/A				
TnT, ng/mL (range)	3.32 (0.062–9.92)	0.008^e^	N/A	0.190	0.190		
K, mmol/L (range)	4.3 (3.0–6.4)	4.5 (3.8–5.3)	4.0 (3.7–4.4)	0.892	0.892	0.642	0.770
Lac, mmol/L (range)	3.3 (1.2–10.8)	1.5^e^	3.2^e^	0.190	0.190	0.132	0.190
T‐Bil, mg/dL (range)	0.6 (0.2–1.3)	0.5 (0.2–0.7)	0.75 (0.5–1.4)	0.261	0.321	0.321	0.321
PT‐INR (range)	1.75 (1.18–3.18)	1.19 (1.12–1.47)^d^	1.22 (0.97–1.56)	0.068	0.102	0.013	0.039
APTT, s (range)	40.7 (29.9–200)	44.0 (36.8–45.1)^d^	34.9 (31.4–46.1)	1.000	1.000	0.148	0.277
Fib, mg/dL (range)	412 (121–741)	241 (184–256)	403 (184–698)	0.362	0.543	0.772	0.772
D‐dimer, μg/mL (range)	3.5 (0.9–33.3)	0.5^e^	1.7 (0.6–2.6)	0.383	0.382	0.003	0.009
Plain radiography^a^	7/7 cases	11/11 cases	6/8 cases				
Cardiothoracic ratio > 55%, *n* (%)	3 (43)	0	0	0.017	0.034	0.038	0.038
ECG	7/7 cases	1/11 cases	0/8 cases				
ST segment changes, *n* (%)	1 (14)	N/A	N/A				
AV block, *n* (%)	5 (71)	N/A	N/A				
Echocardiography	7/7 cases	1/11 cases	1/8 cases				
LVEDd > 110% of normal, *n* (%)	2 (29)	N/A	N/A				
LVEF < 55%, *n* (%)	6 (86)	N/A	N/A				
Pericardial effusion, *n* (%)	6 (86)	N/A	N/A				
Atrioventricular regurgitation, *n* (%)	7 (100)	N/A	N/A				

*Note:* FDR‐adjusted *p*‐values were calculated using the Benjamini–Hochberg procedure.

Abbreviations: AGE, acute gastroenteritis; ALT, alanine aminotransferase; AMC, acute myocarditis; AST, aspartate aminotransferase; BNP, brain natriuretic peptide; CK, creatine kinase; CK‐MB, creatine kinase‐myocardial band; CRP, C‐reactive protein; ECG, electrocardiogram; LDH, lactate dehydrogenase; LVEDd, left ventricular end‐diastolic diameter; LVEF, left ventricular ejection fraction; N/A, not applicable; TnT, troponin‐T; WBC, white blood cells.

^a^ Including thoracic and abdominal imaging.

^b^ Described only case.

^c^ Described only two cases.

^d^ Measured only three cases.

^e^ Measured only.

### 3.3 Multivariable Correlation Analysis

In the present study, there was an age difference between patients with AMC and those with AGE. As both age and LDH demonstrated non‐normal distributions (*p* = 0.0024 and *p* < 0.0001, respectively), Spearman's rank correlation coefficient was calculated. The resulting coefficient (ρ) was 0.043, indicating no correlation between the two variables (*p* = 0.833) (Figure S2). We also performed multivariate correlation analyses for other parameters. LDH levels showed the strongest positive association with AMC (Figure S3).

### 3.4 Receiver Operating Characteristic Curve Analysis for Predicting AMC

**FIGURE 1:**
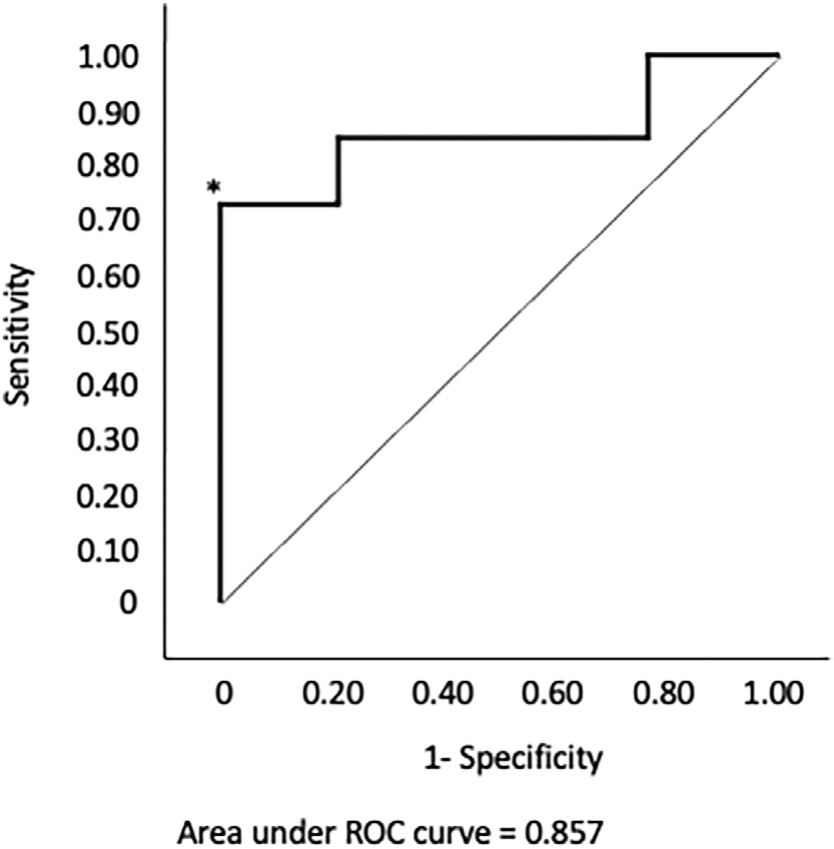
ROC curve illustrating the association between acute myocarditis (AMC) and serum lactate dehydrogenase (LDH) levels in a pediatric population (*n* = 26), comprising seven patients with AMC and 19 patients with acute gastroenteritis (AGE). AGE, acute gastroenteritis; AMC, acute myocarditis; AUC, area under the curve; LDH, lactate dehydrogenase; ROC, receiver operating characteristic; *LDH = 459 U/L.

We found that LDH had the potential to become a trigger for suspicion of AMC. The AUC for LDH was 0.857 (Figure 1). The closer the ROC curve approaches the upper left corner (representing 100% sensitivity and 100% specificity), the better the test performance. We have defined the optimal cutoff value as the point that maximizes the sum of sensitivity and specificity (Youden's index). Setting the LDH level at 459 U/L as the cutoff value yielded a sensitivity of 71% and a specificity of 100%. We also performed ROC curve analysis for the WBC count and CRP, CK, AST, and ALT levels (Figure S4) and presented the 95% confidence intervals for the AUC, sensitivity, and specificity of each biomarker in Table S3. Among the biomarkers examined, AST and ALT showed AUC values relatively close to that of LDH. We performed a statistical comparison of the AUC values using the DeLong test and additionally provided the corresponding confidence intervals (Table S3). However, the DeLong test demonstrated no statistically significant differences between the AUC values of LDH and those of AST or ALT. We performed additional ROC curve analyses excluding fulminant myocarditis. In these analyses, LDH showed the highest AUC (0.737) among the evaluated biomarkers. The results are presented in (Figure S5).

## 4 Discussion

We retrospectively compared the clinical characteristics of patients with AMC with those of patients with AGE. As previously described, the initial symptoms of AMC are similar to those of AGE, making early diagnosis challenging. In this study, preliminary findings were obtained regarding LDH as a trigger for suspicion of AMC in the pediatric population, with high sensitivity (71%) and specificity (100%) when a cutoff value of 459 U/L was used.

Previous reports have highlighted useful diagnostic markers for AMC, such as BNP, CK, and TnT [9]. However, these markers are typically tested in patients with suspected cardiac conditions. For instance, if an initial evaluation is conducted by a physician who is unfamiliar with AMC, troponin or BNP levels may not be measured at the time of the first visit. Similarly, a report from Poland on perimyocarditis noted that BNP levels were not measured in all patients upon admission [13]. Because the initial symptoms of AMC are often nonspecific gastrointestinal complaints, missed or delayed diagnosis remains a significant challenge in pediatric primary care settings [14]. In our study, all patients with AMC were initially diagnosed with AGE or common cold, and these markers were not checked at a previous hospital or in patients with AGE. By contrast, LDH is a routine biochemical test that is often included in the initial laboratory workup in ED and can be performed outside regular hours. LDH, the marker we focused on this time, is an enzyme that the body uses during the process of converting sugar into energy for our cells to use. It is found in many tissues and organs, including the muscles, liver, heart, pancreas, kidneys, brain, and blood cells. Although an LDH test alone cannot show what is damaging to tissues or where the damage is located, it is commonly used in daily clinical practice to assess the severity or progression of tissue damage or diseases. We have summarized the presence or absence of comorbid conditions for each case in the AMC group in Table S4. Importantly, no comorbidities or alternative clinical conditions that could account for elevated LDH levels, other than myocarditis itself, were identified in these cases. The normal range of serum LDH varies slightly with age. Infants and young children usually have much higher normal levels compared to older children and adults. According to Mayo Clinic Laboratories, the age‐specific reference ranges for LDH are as follows: children under 12 months, 180–435 U/L; 1–3 years, 160–370 U/L; 4–6 years, 145–345 U/L; 7–9 years, 143–290 U/L; 10–12 years, 120–293 U/L; 13–15 years, 110–283 U/L; 16–17 years, 105–233 U/L; and adults ≥ 18 years, 122–222 U/L [15]. According to reference ranges from other institutions, the upper limit of LDH for children aged 2–7 years is 386 U/L (as indicated by CHOP Laboratories). Compared to the upper limits of these age‐specific normal values, the cutoff value of 459 U/L calculated in this study was above these limits, suggesting that it did not affect the differences in the normal range across age groups. Moreover, additional analyses excluding fulminant myocarditis were presented in Figure S5. In these analyses, LDH showed the highest AUC (0.737) among the evaluated biomarkers. LDH may be useful even in relatively mild or early‐stage cases, rather than merely reflecting severe systemic injury associated with fulminant disease. Among the biomarkers examined, AST and ALT showed AUC values relatively close to that of LDH. AST and ALT, similar to LDH, are biomarkers that can be influenced by systemic hypoperfusion or ischemic hepatitis during shock states, supporting the possibility that elevated LDH levels in myocarditis may reflect non‐specific increases due to generalized tissue injury. However, one possible explanation for why LDH may be useful as a diagnostic discriminator for myocarditis, rather than as a general severity marker such as AST and ALT, is the difference in the biological half‐lives of these enzymes. Previous studies have reported that, in patients with acute myocardial infarction, the time required for levels to return to the normal range is longer for LDH than AST and CK (12, 5, and 3 days, respectively) [16]. A longer half‐life may extend the detection window, thereby increasing the likelihood of identifying abnormal values during clinical evaluation. Taken together, although the differences in AUC were not statistically significant, LDH demonstrated the highest AUC among the evaluated biomarkers, and its longer half‐life may contribute to improved diagnostic detectability.

In this study, we propose that LDH may serve as a practical and readily available trigger to prompt consideration of myocarditis when cardiac biomarkers have not yet been measured during the early stage of clinical evaluation. However, there was an age discrepancy between the patients with AMC and those with AGE. To evaluate the effect of age on LDH levels, we conducted correlation (Figure S2) and multivariate correlation analyses for the other parameters (Figure S3). Based on these analyses, we conclude that age discrepancy did not affect the LDH results. Although the present study is limited by a small sample size and therefore has an exploratory nature, our findings suggest that LDH could serve as a convenient and promising diagnostic trigger for differentiating AMC from AGE at an early stage. In addition, plain radiography, but not ECG or echocardiography, was performed in almost all patients with AGE. Cardiomegaly showed low sensitivity (48%) but high specificity (100%) in the diagnosis of AMC. Hence, radiography, along with LDH measurement, could also be helpful for the early differential diagnosis of AMC from AGE. However, caution should be exercised when interpreting radiographs. Several studies have reported cases of AMC in which cardiomegaly and pulmonary congestion were not apparent [17, 18]. Patients with AMC, even in cases of cardiogenic shock, do not always have cardiomegaly due to right ventricular dominant myocarditis [17]. These reports emphasize that myocarditis cannot be ruled out on the basis of radiographic findings alone.

Based on previous studies, physical findings have proven useful for the early diagnosis of AMC [9]. Although fever, abdominal pain, and vomiting are also observed in patients with AGE, tachycardia, gallop rhythms, tachypnea, and pale skin are relatively specific signs of AMC. In our study, more than half of the patients exhibited these findings. Particularly, gallop rhythms had high sensitivity (71%) and specificity (100%) in the diagnosis of AMC. Physical findings can be promptly obtained with relatively little effort compared to physiological tests and can precisely reflect circulatory insufficiency. Clinicians are recommended to check the presence of a gallop rhythm when evaluating patients with gastrointestinal symptoms.

This study had several limitations. First, this was a Japanese single‐center retrospective study with a small sample size. Second, the clinical backgrounds of patients were not completely matched. Third, not all patients underwent endomyocardial biopsy (EMB) or cardiac magnetic resonance (CMR) for diagnosis of AMC. EMB or CMR are considered the gold standard to confirm a clinical diagnosis of myocarditis. However, for patients with AMC, due to the severe condition, the risks associated with cardiac catheterization or general anesthesia, and the inconvenience of the examinations, the clinical applications of EMB or CMR are greatly limited [19, 20]. Hence, we diagnosed AMC based on clinical findings, such as signs and symptoms of acute cardiac dysfunction, elevated troponin, or echocardiographic evidence of ventricular dysfunction. Despite these limitations, this study provides new insights into the early diagnosis and subsequent improvement in the prognosis of AMC.

In conclusion, the most clinically meaningful and educationally valuable aspect of this study lies in the diagnostic challenge posed by the overlap of the early symptoms of AMC and AGE. Although generalizability is limited owing to the specific clinical setting, we believe that serum LDH has the potential to be a trigger for suspicion of AMC. A combination of gallop rhythms, cardiomegaly on plain radiography, and LDH may be promising for the early diagnosis of AMC. Notably, LDH is a valuable biomarker because it is not dependent on the skill of medical practitioners. Further, larger prospective studies are required to establish a reliable diagnostic tool for AMC.

## Author Contributions

T.M. was responsible for the conception and design of the study, as well as the acquisition, analysis, and interpretation of data. S.O. was the chief investigator and responsible for the data analysis. Y.O. and T.F. were responsible for acquisition of data. S.H. was responsible for the final approval of the manuscript to be submitted. All authors read and approved the final manuscript.

## Funding

The authors have nothing to report.

## Disclosure

The authors have nothing to report.

## Ethics Statement

This study was approved by the Institutional Review Board of the Yamaguchi University Hospital (No. H2023‐152).

## Consent

Written informed consents were obtained from patients' parents.

## Conflicts of Interest

The authors declare no conflicts of interest.

## Supporting information


**Figure S1:** Comparison of box plots of serum LDH in three groups. AMC, acute myocarditis; LDH, lactate dehydrogenase.

**Figure S2:** Scatter plot illustrating the association between serum lactate dehydrogenase (LDH) and age. Black and white circles indicate AGE group and AMC group, respectively. AGE, acute gastroenteritis; AMC, acute myocarditis; LDH, lactate dehydrogenase.

**Figure S3:** Multivariate correlation analysis illustrating the association between acute myocarditis (AMC) and biomarkers. ALT, alanine aminotransferase; AST, aspartate aminotransferase; CK, creatine kinase; CRP, C‐reactive protein; K, potassium; LDH, lactate dehydrogenase; T‐Bil, total bilirubin; WBC, white blood cells.

**Figure S4:** ROC curves illustrating the association between acute myocarditis (AMC) and the levels of several biomarkers in a pediatric population (*n* = 26) comprising seven patients with AMC and 19 patients with acute gastroenteritis (AGE). AGE, acute gastroenteritis; ALT, alanine aminotransferase; AMC, acute myocarditis; AST, aspartate aminotransferase; AUC, area under the curve; CK, creatine kinase; CRP, C‐reactive protein; ROC, receiver operating characteristic; WBC, white blood cells; * cutoff value in each graph.

**Figure S5:** ROC curves illustrating the association between AMC without fulminant myocarditis and the levels of several biomarkers in a pediatric population (*n* = 22) comprising three patients with non‐fulminant AMC and 19 patients with acute gastroenteritis (AGE). AGE, acute gastroenteritis; ALT, alanine aminotransferase; AMC, acute myocarditis; AST, aspartate aminotransferase; AUC, area under the curve; CK, creatine kinase; ROC, receiver operating characteristic.

**Table S1:** Clinical characteristics of patients with acute gastroenteritis.

**Table S2:** Clinical presentations by myocarditis type.

**Table S3:** Comparison of AUCs using DeLong test.

**Table S4:** Comorbidities in Individual Patients with AMC.

## Data Availability

The data that support the findings of this study are available on request from the corresponding author. The data are not publicly available due to privacy or ethical restrictions.
