# Supplementary figures and images for: Early Detection of Acute Myocarditis in the Pediatric Population Using Clinically Accessible Data
Source: Pediatr Int. 2026 Jul 27;68(1):e70492. doi: 10.1111/ped.70492 (PMC13403099; doi:10.1111/ped.70492)

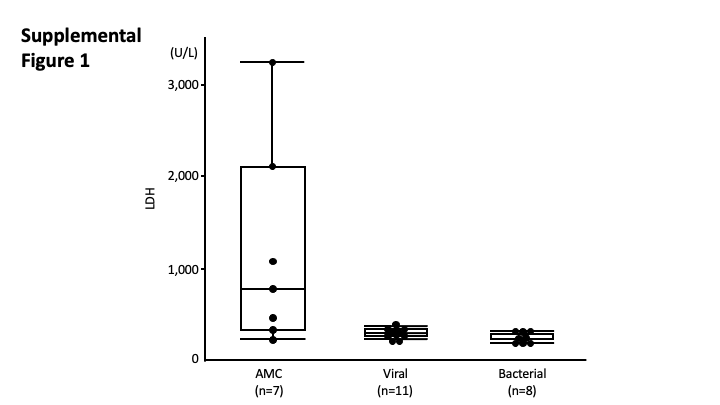

Supplement: Supplementary file 1 — Figure S1: Comparison of box plots of serum LDH in three groups. AMC, acute myocarditis; LDH, lactate dehydrogenase. [file PED-68-e70492-s008.tiff]

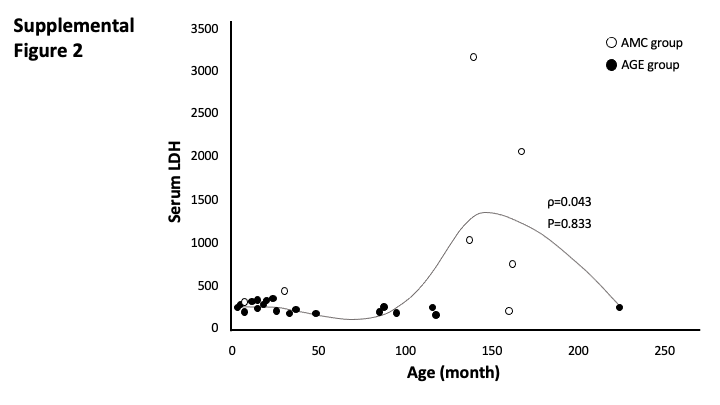

Supplement: Supplementary file 2 — Figure S2: Scatter plot illustrating the association between serum lactate dehydrogenase (LDH) and age. Black and white circles indicate AGE group and AMC group, respectively. AGE, acute gastroenteritis; AMC, acute myocarditis; LDH, lactate dehydrogenase. [file PED-68-e70492-s003.tiff]

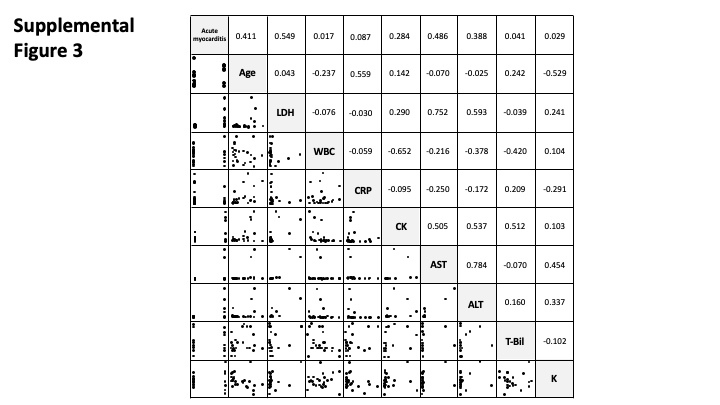

Supplement: Supplementary file 3 — Figure S3: Multivariate correlation analysis illustrating the association between acute myocarditis (AMC) and biomarkers. ALT, alanine aminotransferase; AST, aspartate aminotransferase; CK, creatine kinase; CRP, C‐reactive protein; K, potassium; LDH, lactate dehydrogenase; T‐Bil, total bilirubin; WBC, white blood cells. [file PED-68-e70492-s002.tiff]

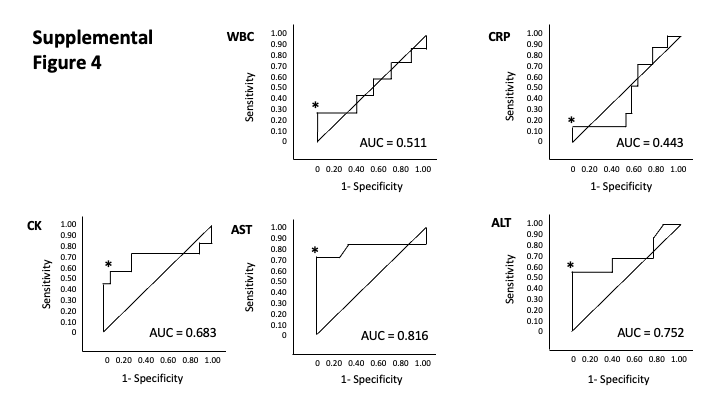

Supplement: Supplementary file 4 — Figure S4: ROC curves illustrating the association between acute myocarditis (AMC) and the levels of several biomarkers in a pediatric population (n = 26) comprising seven patients with AMC and 19 patients with acute gastroenteritis (AGE). AGE, acute gastroenteritis; ALT, alanine aminotransferase; AMC, acute myocarditis; AST, aspartate aminotransferase; AUC, area under the curve; CK, creatine kinase; CRP, C‐reactive protein; ROC, receiver operating characteristic; WBC, white blood cells; * cutoff value in each graph. [file PED-68-e70492-s006.tiff]

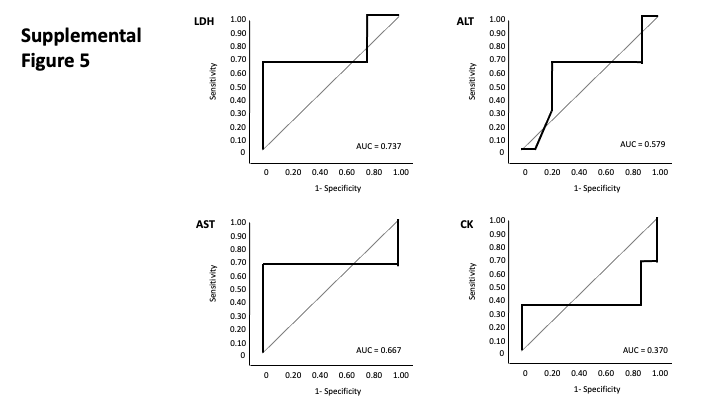

Supplement: Supplementary file 5 — Figure S5: ROC curves illustrating the association between AMC without fulminant myocarditis and the levels of several biomarkers in a pediatric population (n = 22) comprising three patients with non‐fulminant AMC and 19 patients with acute gastroenteritis (AGE). AGE, acute gastroenteritis; ALT, alanine aminotransferase; AMC, acute myocarditis; AST, aspartate aminotransferase; AUC, area under the curve; CK, creatine kinase; ROC, receiver operating characteristic. [file PED-68-e70492-s007.tiff]
