# Supplementary material for: Early Detection of Acute Myocarditis in the Pediatric Population Using Clinically Accessible Data
Source: Pediatr Int. 2026 Jul 27;68(1):e70492. doi: 10.1111/ped.70492 (PMC13403099; doi:10.1111/ped.70492)
Supplement: Supplementary file 6 — Table S1: Clinical characteristics of patients with acute gastroenteritis. [file PED-68-e70492-s004.docx]

**Supplemental Table 1. Clinical characteristics of patients with acute gastroenteritis**

| Total number | | | 19 |
| --- | --- | --- | --- |
| Viral enteritis, n (%) | | | 11 (58) |
|  | Sex (male/female), n | | 5/6 |
|  | Causes, n | Norovirus  Rotavirus  undetected | 5  5  1 |
| Bacterial enteritis, n (%) | | | 8 (42) |
|  | Sex (male/female), n | | 3/5 |
|  | Causes, n | *Salmonella spp.*  *Campylobacter spp.*  *Enteropathogenic Escherichia coli (O-157)* | 3  2  3 |
