# Supplementary material for: Early Detection of Acute Myocarditis in the Pediatric Population Using Clinically Accessible Data
Source: Pediatr Int. 2026 Jul 27;68(1):e70492. doi: 10.1111/ped.70492 (PMC13403099; doi:10.1111/ped.70492)
Supplement: Supplementary file 7 — Table S2: Clinical presentations by myocarditis type. [file PED-68-e70492-s001.docx]

**Supplemental Table 2. Clinical presentations by myocarditis type**

|  | Fulminant (n=4) | Acute (n=3) | P value | |
| --- | --- | --- | --- | --- |
| Female, n (%) | 2 (50) | 2 (67) | 1.000 | |
| Age, month (IQR) | 153 (39-168) | 137 (29-162) | 0.629 | |
| Initial symptoms before admission |  |  |  | |
| Fever, n (%) | 2 (50) | 3 (100) | 0.429 | |
| Abdominal Pain, n (%) | 2 (50) | 2 (67) | 1.000 | |
| Nausea, n (%) | 2 (50) | 1 (33) | 1.000 | |
| Vomiting, n (%) | 3 (75) | 2 (67) | 1.000 | |
| Diarrhea, n (%) | 1 (25) | 1 (33) | 1.000 | |
| Fatigue, n (%) | 2 (50) | 0 (0) | 0.429 | |
| Appetite loss, n (%) | 1 (25) | 2 (67) | 0.486 | |
| Convulsion, n (%) | 1 (25) | 0 (0) | 1.000 | |
| Palpitation, n (%) | 1 (25) | 0 (0) | 1.000 | |
| Chest pain, n (%) | 1 (25) | 0 (0) | 1.000 | |
| Respiratory distress, n (%) | 2 (50) | 1 (33) | 1.000 | |
| Headache, n (%) | 2 (50) | 0 (0) | 0.429 | |
| Nasal mucus, n (%) | 1 (25) | 1 (33) | 1.000 | |
| Cough, n (%) | 1 (25) | 2 (67) | 0.486 | |
| Physical examination findings at admission | |  |  | |
| Tachypnea, n (%) | 2 (50) | 2 (67) | 1.000 | |
| Retraction breathing, n (%) | 1 (25) | 0 (0) | 1.000 | |
| Tachycardia, n (%) | 3 (75) | 3 (100) | 1.000 | |
| Poor skin turgor, n (%) | N/A | N/A |  | |
| Peripheral coldness, n (%) | 2 (50) | 1 (33) |  | |
| Pale skin, n (%) | 2 (50) | 2 (67) |  | |
| Abdominal distention, n (%) | 0 (0) | 0 (0) |  | |
| Abdominal tenderness, n (%) | 0 (0) | 0 (0) |  | |
| Edema, n (%) | 1 (25) | 0 (0) | 1.000 | |
| Gallop rhythms, n (%) | 3 (75) | 2 (67) | 1.000 | |
| Cardiac murmur, n (%) | 4 (100) | 3 (100) |  | |
| Intact survival, n (%) | 1 (25) | 2 (67) | 0.486 | |
| Death, n (%) | 1 (25) | 0 (0) | 1.000 | |
| Blood Examination |  |  |  | |
| WBC, /μL (range) | 9,455 (4,480-11,850) | 19,810 (6,720-19,860) | 0.400 | |
| CRP, mg/dL (range) | 2.08 (0.01-5.78) | 3.66 (2.72-16.8) | 0.400 | |
| AST, U/L (range) | 971 (15-2,495) | 75 (15-101) | 0.229 | |
| ALT, U/L (range) | 1,041 (11-2,227) | 12 (11-62) | 0.057 | |
| LDH, U/L (range) | 1,449 (236-3,234) | 459 (236-1,085) | 0.400 | |
| CK, U/L (range) | 941 (122-1,879) | 46 (25-1,513) | 0.400 |  |
| CK-MB, U/L (range) | 66 (32-127) | 21.5 (7-22) | 0.057 |  |
| BNP, pg/mL (range) | 1,530 (550-6,485) | 914 (639-1,120) | 1.000 |  |
| TnT, ng/mL (range) | 5.02 (0.06-9.92) | 0.069 (0.062-3.65) | 0.229 |  |
| K, mmol/L (range) | 4.6 (3.4-6.4) | 4.3 (3.0-5.4) | 0.860 |  |
| Lac, mmol/L (range) | 5.7 (3.3-10.8) | 2.6 (1.2-2.7) | 0.052 |  |
| T-Bil, mg/dL (range) | 1.05 (0.6-1.3) | 0.4 (0.2-0.5) | 0.052 |  |
| PT-INR (range) | 2.18 (1.62-3.16) | 1.55 (1.18-1.75) | 0.112 |  |
| APTT, s (range) | 54.1 (35.3-200) | 38.9 (29.9-40.7) | 0.216 |  |
| Fib, mg/dL (range) | 194 (121-452) | 475 (412-741) | 0.112 |  |
| D-dimer, μg/mL (range) | 12.3 (1.1-33.3) | 2.1 (0.9-3.5) | 0.216 |  |
| Plain radiography^*^ | 4/4 cases | 3/3 cases |  |  |
| cardiothoracic ratio > 55%, n (%) | 2 (50) | 1 (33) | 1.000 |  |
| ECG | 4/4 cases | 3/3 cases |  |  |
| ST segment changes, n (%) | 1 (25) | 0 (0) | 1.000 |  |
| AV block, n (%) | 3 (75) | 2 (67) | 1.000 |  |
| Echocardiography | 4/4 cases | 3/3 cases |  |  |
| LVEDd > 110% of normal, n (%) | 2 (50) | 0 (0) | 0.429 |  |
| LVEF < 55%, n (%) | 3 (75) | 3 (100) | 1.000 |  |
| Pericardial effusion, n (%) | 3 (75) | 3 (100) | 1.000 |  |
| Atrioventricular regurgitation, n (%) | 4 (100) | 3 (100) |  |  |

*including thoracic and abdominal imaging，AGE indicates acute gastroenteritis; AMC, acute myocarditis; ALT, alanine aminotransferase; AST, aspartate aminotransferase; BNP, brain natriuretic peptide; CK, creatine kinase; CK-MB, creatine kinase-myocardial band; CRP, C-reactive protein; ECG, electrocardiogram; LDH, lactate dehydrogenase; LVEDd, left ventricular end-diastolic diameter; LVEF, left ventricular ejection fraction; N/A, not applicable; TnT, troponin-T; WBC, white blood cells.
