# Supplementary material for: Early Detection of Acute Myocarditis in the Pediatric Population Using Clinically Accessible Data
Source: Pediatr Int. 2026 Jul 27;68(1):e70492. doi: 10.1111/ped.70492 (PMC13403099; doi:10.1111/ped.70492)
Supplement: Supplementary file 8 — Table S3: Comparison of AUCs using DeLong test. [file PED-68-e70492-s009.docx]

**Supplemental Table 3. Comparison of AUCs using DeLong test.**

|  | AUC (95% CI) | Cutoff value | P value (vs. LDH) | Sensitivity (95% CI) | Specificity (95% CI) |
| --- | --- | --- | --- | --- | --- |
| LDH | 0.875 (0.634-1.000) | 459 | ref. | 0.714 (0.290-0.963) | 1.000 (0.751-1.000) |
| AST | 0.816 (0.536-1.000) | 75 | 0.265 | 0.714 (0.290-0.963) | 1.000 (0.741-1.000) |
| ALT | 0.752 (0.458-1.000) | 62 | 0.393 | 0.714 (0.290-0.963) | 0.895 (0.669-0.987) |
| CK | 0.683 (0.353-1.000) | 520 | 0.189 | 0.571 (0.184-0.901) | 0.944 (0.727-0.999) |
| WBC | 0.511 (0.205-0.817) | 1,9810 | 0.003 | 0.286 (0.037-0.710) | 1.000 (0.751-1.000) |
| CRP | 0.444 (0.187-0.701) | N/A | N/A | N/A | N/A |

Statistical significance was assessed using the DeLong test with LDH as the reference. ALT, alanine aminotransferase; AST, aspartate aminotransferase; AUC, area under the curve; CI, confidence interval; CK, creatine kinase; CRP, C-reactive protein; LDH, lactate dehydrogenase; N/A, not applicable; ref. , reference; WBC, white blood cells.
