# Supplementary material for: Early Detection of Acute Myocarditis in the Pediatric Population Using Clinically Accessible Data
Source: Pediatr Int. 2026 Jul 27;68(1):e70492. doi: 10.1111/ped.70492 (PMC13403099; doi:10.1111/ped.70492)
Supplement: Supplementary file 9 — Table S4: Comorbidities in Individual Patients with AMC. [file PED-68-e70492-s005.docx]

**Supplemental Table 4. Comorbidities in Individual Patients with AMC**

| Case No. | Type of AMC | Age | Sex | LDH  (U/L) | Comorbidities | | | | |
| --- | --- | --- | --- | --- | --- | --- | --- | --- | --- |
|  |  |  |  |  | Seizure | Skeletal muscle injury | Severe dehydration | Hepatic injury  unrelated to myocarditis | Malignant tumor |
| 1 | Fulminant | 14y | F | 2,106 | No | No | No | No | No |
| 2 | Fulminant | 13y | M | 792 | No | No | No | No | No |
| 3 | Fulminant | 11y | F | 3,234 | No | No | No | No | No |
| 4 | Fulminant | 5m | M | 332 | No | No | No | No | No |
| 5 | Acute | 13y | F | 236 | No | No | No | No | No |
| 6 | Acute | 2y | F | 459 | No | No | No | No | No |
| 7 | Acute | 11y | M | 1,085 | No | No | No | No | No |

ALT, alanine aminotransferase; AMC, acute myocarditis; AST, aspartate aminotransferase; AUC, area under the curve; CK, creatine kinase; CRP, C-reactive protein; F, female; LDH, lactate dehydrogenase; m, month; M, mail, N/A, not applicable, y, year; WBC, white blood cells.
